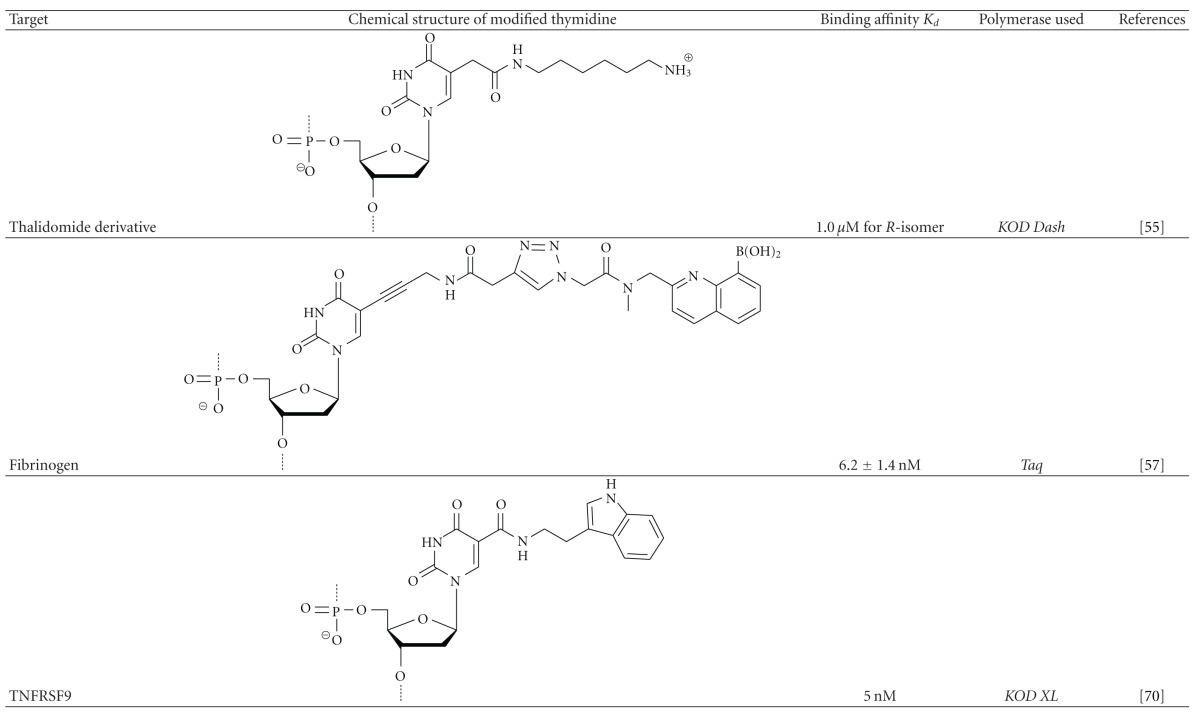# Artificial Specific Binders Directly Recovered from Chemically Modified Nucleic Acid Libraries

**DOI:** 10.1155/2012/156482

**Published:** 2012-10-08

**Authors:** Yuuya Kasahara, Masayasu Kuwahara

**Affiliations:** Graduate School of Engineering, Gunma University, 1-5-1 Tenjin-cho, Kiryu 376-8515, Japan

## Abstract

Specific binders comprised of nucleic acids, that is, RNA/DNA aptamers, are attractive functional biopolymers owing to their potential broad application in medicine, food hygiene, environmental analysis, and biological research. Despite the large number of reports on selection of natural DNA/RNA aptamers, there are not many examples of direct screening of chemically modified nucleic acid aptamers. This is because of (i) the inferior efficiency and accuracy of polymerase reactions involving transcription/reverse-transcription of modified nucleotides compared with those of natural nucleotides, (ii) technical difficulties and additional time and effort required when using modified nucleic acid libraries, and (iii) ambiguous efficacies of chemical modifications in binding properties until recently; in contrast, the effects of chemical modifications on biostability are well studied using various nucleotide analogs. Although reports on the direct screening of a modified nucleic acid library remain in the minority, chemical modifications would be essential when further functional expansion of nucleic acid aptamers, in particular for medical and biological uses, is considered. This paper focuses on enzymatic production of chemically modified nucleic acids and their application to random screenings. In addition, recent advances and possible future research are also described.

## 1. Introduction

RNA/DNA aptamers, which are specific for a broad spectrum of targets, can be artificially created by systematic evolution of ligands by exponential enrichment (SELEX) methods [1, 2]. Large-scale chemical synthesis of RNA/DNA aptamers is possible, and synthesizing them is less expensive than producing antibodies; therefore, they have been considered as alternatives to therapeutic antibodies. Although RNA/DNA aptamers do not cause antibody-dependent cell-mediated cytotoxicity (ADCC) and complement-dependent cytotoxicity (CDC), their specific binding abilities are expected to neutralize actions on the target and relieve symptoms. Indeed, the first example of an aptamer drug, “Macugen (pegaptanib sodium injection)” is being used for age-related macular degeneration (AMD) therapy [3]. Pegaptanib is a RNA-based aptamer that involves 2′-fluoropyrimidine nucleotides (U, C) and 2′-methoxy purine nucleotides (A, G) to remain intact under physiological conditions. In addition, a branched polyethylene glycol strand (40 kDa) and 3′-thymidylic acid are introduced at its 5′ and 3′ ends, respectively. The 5′-end modification is known to prolong circulation time *in vivo* as well as to enhance nuclease resistance. Pegaptanib tightly binds to the vascular endothelial growth factor (VEGF) in a Ca^2+^-dependent fashion with a dissociation constant (*K*_*d*_) of 200 pM, while the corresponding aptamer, which lacks the 5′- and 3′-end capping, has much higher affinity (*K*_*d*_ = 49 ± 6 pM at 37°C in phosphate-buffered saline containing 2 mM Ca^2+^). Incidentally, the *K*_*d*_ value of the anti-VEGF antibody, “Avastin (bevacizumab),” which is used for cancer therapies, is 1.1 nM at 25°C. The natural type of anti-VEGF RNA aptamers also shows high-binding affinity at a picomolar range (*K*_*d*_ = 140 ± 4 pM at 37°C in phosphate-buffered saline containing no Ca^2+^) [4], indicating that the effects of chemical modifications on binding affinity are not significant, considering the different Ca^2+^ concentrations used. In contrast, the effects on biostability are remarkable; pegaptanib was found to be stable after incubation at ambient temperature for 18 h in human plasma containing ethylenediaminetetraacetic acid, whereas unmodified oligoribonucleic acids are known to degrade within a few minutes *in vivo* [5].

**Figure 1:**
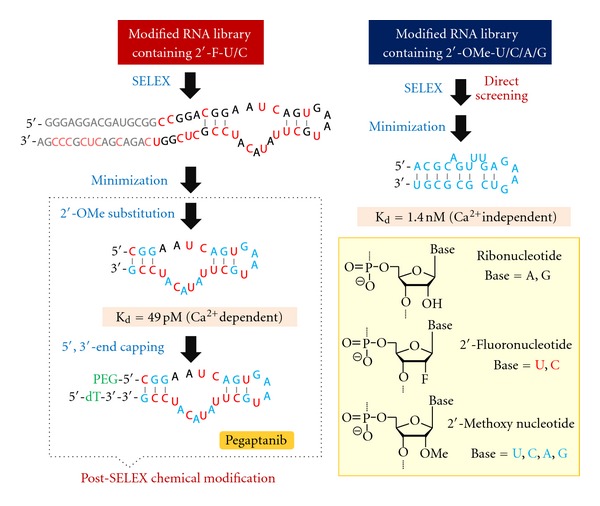
Preparation scheme for chemically modified nucleic acid aptamers that bind to VEGF. High nuclease-resistant 2′-methoxy nucleotides were introduced through Post-SELEX modification process (left), and fully modified 2′-OMe RNA aptamers were directly selected from a library of 2′-OMe transcripts (right).

Owing to the limited tolerance for modified substrates of the RNA polymerase (T7 RNA polymerase) used for SELEX, the 2′-methoxy (–OMe) groups need to be replaced with 2′-hydroxy (–OH) groups of natural purine nucleotides after obtaining the precursor from a modified RNA library involving 2′-fluoro (–F) analogs of uridine and cytidine and natural adenosine and guanosine (Figure 1). The post-SELEX modifications have been successful in rendering nuclease resistance but required considerable time and effort because binding affinities could be markedly decreased or eliminated, depending on the position of the replacement. To overcome this problem, T7 RNA polymerase double-mutant Y639F/H784A was used for enzymatic preparation of the modified RNA library in the SELEX processes, and 2′-OMe RNA aptamers specific to VEGF have been successfully screened directly [6]. One of the 2′-OMe RNA aptamers that could be minimized to 23-mer (which is an unusual short length) was found to be quite stable, and no degradation was observed after incubation at 37°C for 96 h in plasma. Despite being successful for direct screening, structural minimizing, and biostability enhancing, these aptamers were found to have binding affinities in a low nanomolar range that were inferior to those of pegaptanib and its precursors.

This may be because the potential binding ability of the chemical library used was inherently low, and/or the unusual polymerase reaction would cause unfavorable critical biases in the sequences of the chemical library constructed. Conversely, it may also be possible that differences in the selection outcomes would not be sufficient to clarify their causes because only a part of all possible sequences were screened. This is a characteristic difficulty in SELEX when chemical modification is involved. Regardless of this difficulty, a polymerase reaction involving modified nucleotides is a key step that should be improved and optimized to construct desirable direct screening systems for modified RNA/DNA aptamers when the SELEX methods are applied.

## 2. Enzymatic Modified RNA/DNA Polymerization

### 2.1. Kinetics of Modified Substrate Triphosphates Incorporation for SELEX

RNA/DNA polymerases incorporate substrate triphosphates (NTPs/dNTPs) corresponding to the type of bases on the template strand and successively add them to the 3′ end of the extending strand to form 3′,5′-phosphodiester linkage. Some polymerases are known to accept chemically modified NTPs/dNTPs as substrates and can produce nucleic acid polymers containing foreign functionalities. Such polymerase reactions are applied to DNA sequencing [7–10], fluorophore, and redox labeling [11, 12], expanding the genetic alphabet [13–15], and preparing library for SELEX [16–48]. Unlike enzymatic functional labeling of DNA, for which modified dNTP is often used in the presence of the corresponding natural dNTP to increase product yields, modified NTP/dNTP is generally used in the absence of the corresponding natural NTP/dNTP when modified nucleic acid libraries are prepared. This is because the natural nucleotide needs to be completely replaced with the corresponding modified nucleotide at all sites incorporated into the extending strand. In general, total replacement could decrease the product yield because the catalytic efficiencies of the polymerase may be affected by the modifications not only for substrate triphosphate but also on the extending strand and template. Previously reported kinetic studies using a base modified nucleotide showed that the reaction efficiencies of single modified nucleotide incorporation are drastically decreased when the modifications exist on the 3′ terminus of the extending strand, although the single incorporation of the modified substrate proceeds smoothly at almost the same rate as the corresponding natural substrate [37]. The results indicate that the successive incorporation of modified nucleotides is the most difficult aspect of strand extension. Therefore, the inefficiency of modified RNA/DNA polymerization could naturally bias the outcomes of the selection; it could unintentionally lead to the exclusion of the sequences with the highest binding affinity. To reduce this influence, reactions are often conducted under very high enzyme and/or substrate concentrations to achieve large reaction velocities. However, it should be noted that such conditions are prone to result in a high frequency of misincorporations. As the solution strategy, polymerase variants, triphosphate analogs, and their combinations that improve the reaction efficiency have been developed and are still being studied. Furthermore, when modified DNA is used in SELEX, the modified DNA is normally amplified indirectly by a polymerase chain reaction (PCR) to prepare the next library. After affinity selection, the selected modified DNA is reverse transcribed and PCR amplified to natural DNA, and then transcribed to modified DNA, even when PCR amplification was available for the modification.

### 2.2. Library Preparation

T7 RNA polymerase and its variants have primarily been used for SELEX using modified RNA. These polymerases could accept NTP analogs of 2′-thio (–SH) [17], 2′-amino (–NH_2_) [19], 2′-azido (–N_3_) [29], 2′-hydroxymethyl (–CH_2_OH) [33], and 4′-thio (–S–) [39] in addition to 2′-F and 2′-OMe as substrates. Furthermore, NTP analogs with base modification (e.g., C5-modified uridines and cytidines) and with phosphate-modified nucleosides (e.g., 5′-(*α*-thio)triphosphates and 5′-(*α*-borano)triphosphates) [21, 30], which are available for modified RNA polymerization, have also been reported. It is known that polymerization by T7 RNA polymerases starts with the generation of purine-rich oligonucleotide with a length of approximately 10 residues in the initiation step, and the composition of the leader oligonucleotide, preferably with guanosine residues, greatly influences the transcript yields [49]. In the initiation step, the polymerases are sensitive to modifications of the 2′-hydroxyl group, while recognition of the 2′-position is tolerated during the elongation step [50]. Therefore, reactions are often performed using modified NTPs plus a low ratio of natural GTP to increase transcript yields. For example, a highly modified 2′-OMe RNA library was provided in sufficient yield by a reaction containing four 2′-OMe NTPs and natural GTP, which was catalyzed by the T7 RNA polymerase variant (Y639F/H784A) and initiated by the formation of the leader oligonucleotide with a sequence of GGGAGAGGAGAGAA [6]. Thus, preparations of modified RNA libraries typically require a low ratio of natural GTP or both GTP and ATP, and polymerase mutation could reduce the ratio.

For SELEX using modified DNA, certain thermophilic DNA polymerases, for example, *Pwo*, *Pfu*, *Vent(exo-)*, *Deep Vent(exo-)*, and *KOD Dash*, that belong to the evolutional family B, were found to be preferable to other types of DNA polymerases [31]. Especially in successive incorporations of modified nucleotides, those polymerases were found to exhibit much superior performance than family A DNA polymerases such as *Taq*, *Tth*, and *thermo sequenase*. In addition, a family D DNA polymerase derived from *Pyrococcus horikoshii* did not show any tolerance for chemical modification in the experiments using C5-substituted pyrimidine nucleoside triphosphates [41]. The efficiency of enzymatic production using modified dNTP varies depending on the site where the substituent is introduced. As for base modification, dNTP analogs with pyrimidine substituted at the 5th position and purine substituted at the 7th position of the base moiety tend to be acceptable for DNA polymerases and act as good substrates [27, 32]. Modified purine nucleotide analogs at the 8th position can also be incorporated but with lower efficiency [10, 42]. In addition, sugar modifications such as 2′-fluoro, 2′-fluoro-D-arabino, and 2′-*O*,4′-*C*-methylene (BNA/LNA) were also found to be acceptable [44–46]. Furthermore, phosphate-modified dNTPs with 5′-(*α*-thio)triphosphates and 5′-(*α*-borano)triphosphates were found to work as alternative substrates. Certain types of DNA polymerases were also found to accept some modifications of the leaving group of the phosphate moiety [9], although those analogs have been applied for advanced DNA sequencing and not for SELEX.

**Figure 2:**
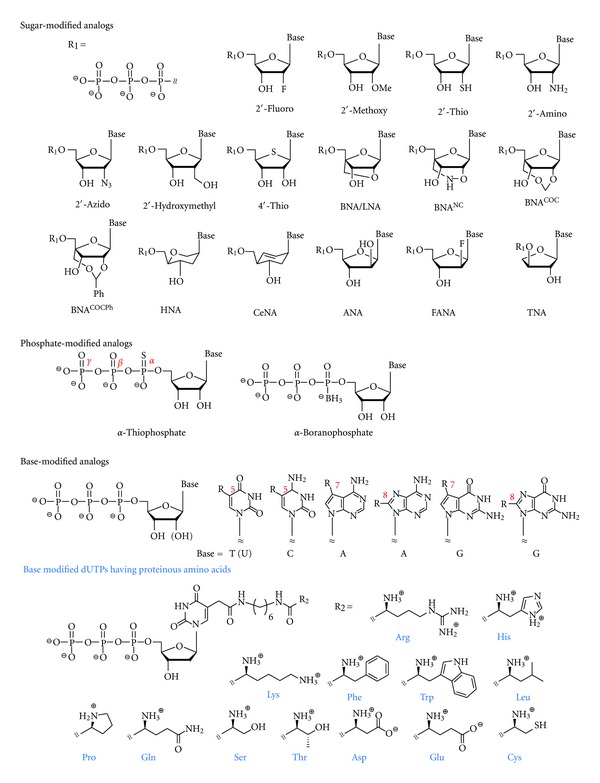
Examples of modified nucleoside triphosphates that act as substrates for polymerase reactions.

Among the modifications, DNA polymerases could endure substitution at the base moieties, and various functional groups could be introduced into those positions with relatively high efficiency. When artificially created protein-like functional nucleic acids were considered, researchers would first envisage introducing proteinous amino acids into nucleic acids. Indeed, base-modified dNTP analogs bearing various proteinous amino acids or their side chains have been reported to date, and their substrate properties in polymerase reactions such as PCR and primer extension have been investigated (Figure 2) [38]. For example, PCR assays using *KOD Dash* DNA polymerase showed that triphosphates containing amino acyl group with basic (Arg, His, Lys), aromatic (Phe, Trp), aliphatic (Leu, Pro), and neutral hydrophilic (Gln, Ser, Thr) side chains act as good substrates, while those with acidic (Asp, Glu) and thiol (Cys) side chains act as poor substrates. Production of DNA-containing cysteinyl residue necessitated the addition of dithiothreitol as a reduction reagent. To introduce plural functionalities with high density, it was found that four natural nucleotides (A, G, C, T) are totally replaced with four base-modified nucleotides by the addition of manganese chloride and betaine [32, 35]. Those additives could improve efficiency and yield in the enzymatic production of modified DNA, although they could raise the frequency of misincorporation at the same time [51, 52].

## 3. Efficacies of Introduced Foreign Functional Groups

### 3.1. Biostability and Binding Property

In modified RNA/DNA aptamers created by direct screening of modified libraries, the effectiveness of modified groups introduced on biostability has been sufficiently supported by many examples. In particular, RNA-based aptamers with modified sugar, in which the 2′-hyrdoxyl group is substituted, dramatically enhanced nuclease resistances. In addition to pegaptanib, for example, a modified RNA aptamer with 2′-NH_2_-U/C specific to human neutrophil elastase (HNE) retained its intact form in serum with a degradation half-life of approximately 20 h, while the corresponding natural RNA mostly degraded just within 5 min [53]. Moreover, thioaptamers, RNA/DNA-based aptamers with modified phosphate, that is, *α*-phosphorothioate, which typically displays increased stability in the biological milieu, have been developed [54]. On the other hand, reports that imply positive effects of modification in binding properties are limited. That is, chemical modifications do not always result in raising binding affinity and specificity as researchers expected (Sections 3.2 and 3.3). Regarding the beneficial aspects of modification, we previously reported that a base-modified DNA aptamer, which was recovered from a modified DNA library containing 5-(2-(6-aminohexylamino)-2-oxoethyl-2′-deoxyuridine, can bind with the R-isomer of a thalidomide derivative with high enantioselectivity [55]. Moreover, another base-modified DNA aptamer, selected from a DNA library containing arginine residues, could clearly distinguish the dicarboxylate moiety of D-glutamic acid from that of L-isomer [56]. Also, Li et al. reported that a base-modified DNA aptamer carrying the boronic acid moiety, which was obtained by implementing thorough counter selections, can sensitively recognize glycosylation sites of fibrinogen as a glycoprotein [57]. Although the impact on binding affinity seems to be limited, these examples merit attention because of their implications for the possibility of expanding binding modes in molecular recognition with introduced foreign functional groups. Thus, the use of base-modified DNA for selection library would certainly be one of the most promising strategies to improve binding properties, because of the aforementioned functional expandability; repertories of modification in bases, which could be available for enzymatic incorporation, are much broader than those in the other parts. Furthermore, the number of candidates for polymerase variants capable of catalyzing modified nucleotide polymerization with high efficiency is expected to be much greater in DNA polymerases than in RNA polymerases. 

### 3.2. Efficacy of Modification on Binding to Small Molecular Targets

To probe superiorities in terms of introducing foreign functionality, random screenings from nucleic acid libraries of compounds having different chemical structures should be performed using the same target, and those selection outcomes should be collated carefully. One small molecular target that has generated significant attention is adenosine-5′-triphosphate (ATP). To date, ATP-binding aptamers have been obtained from libraries of RNA, DNA, modified RNA, and modified DNA [58–62]. However, neither the benefit of modification nor the contribution expected from the chemical structure of foreign functionality to binding affinity and specificity is likely to be substantiated by those SELEX experiments (Table 1).

RNA aptamers were reported by Sazani et al. and Sassanfar and Szostak, respectively, and a modified RNA aptamer was reported by Vaish et al.; those aptamers were found to bind to ATP in a 1 : 1 stoichiometry, depending on the Mg^2+^ concentration. The *K*_*d*_ value of Sassanfar's RNA aptamer was found to be 6–8 *μ*M at 5 mM Mg^2+^ and 0.7 *μ*M at 20 mM Mg^2+^, while that of Sazani's RNA aptamer was 11 *μ*M at 10 mM Mg^2+^ and 4.8 *μ*M at 30 mM Mg^2+^. The modified RNA aptamer contains 5-(3-aminopropyl) uridine, and its binding affinity was significantly improved as the Mg^2+^ concentration increased up to 3 mM, with a *K*_*d*_ value estimated to be between 0.45 and 1.1 *μ*M at 6 mM Mg^2+^. Although there were some differences in the selection protocol, no notable differences in binding affinities between natural and modified RNA aptamers were observed, with *K*_*d*_ values for the modified RNA aptamers ranging from approximately 10^−7^ to 10^−6^.

Electrostatic interactions between the introduced amino group and the triphosphate moiety of ATP were expected; however, the modified RNA aptamer does not clearly distinguish ATP from ADP, AMP, or adenosine (*K*_*d*_ = 1.02, 1.01, 2.18, and 3.91 *μ*M, resp.) under the same conditions, although the foreign functional group is essential for target binding. Similarly, significant binding specificities to the triphosphate moiety in Sassanfar's RNA were not observed, with *K*_*d*_ values for ATP and those related targets being similar within the range of 2-3 *μ*M. In contrast, surprisingly, Sazani's RNA aptamer exhibits superior binding specificity for the moiety; its binding affinity is 64-fold lower because of a lack of *γ*-phosphate in ATP and is 1100-fold lower because of lack of both *β*- and *γ*-phosphates. These results indicate that the introduction of a cationic functional group could be alternated with that of native functionalities in RNA and divalent metal ions in solution. Conversely, the modified RNA aptamer exhibits superior specificity for the base moiety in comparison to Sazani's RNA aptamer; the former shows 150-fold weaker binding to inosine-5′-triphosphate (ITP) and does not bind to GTP, CTP, or UTP, while the latter shows 66-, 19-, 680-, and 610-fold weaker binding to ITP, GTP, CTP, and UTP, respectively. 

A DNA aptamer reported by Huizenga and Szostak binds to ATP with an apparent *K*_*d*_ value of 6 ± 3 *μ*M, which was calculated assuming a 1 : 1 binding ratio and shows a similar affinity to AMP and adenosine. However, a sequence containing a representative motif of Huizenga's DNA aptamer was found to cooperatively bind two ATP molecules with a binding ratio of 1 : 2 and a *K*_*d*_ of 9 ± 2 *μ*M^2^. Interestingly, a modified DNA aptamer containing 5-(3-aminopropyl)-2′-deoxyuridine also forms a 1 : 2 complex with ATP, with a *K*_*d*_ value of 6 ± 1 *μ*M^2^. Unlike the ATP-binding-modified RNA aptamer, the DNA aptamer with thymidine replacing the modified dU was found to retain the binding affinity, although the affinity for ATP was lowered by approximately 2-fold (*K*_*d*_ = 13 ± 4 *μ*M^2^). This indicates that the introduction of functionality did not dramatically influence the structures or activities of the aptamers selected. 

### 3.3. Efficacy of Modifications on Binding to Protein Targets

Thrombin (FIIa) is a multifunctional serine protease that plays key roles in hemostasis, thrombosis, and inflammation. Therefore, developing nucleic acid aptamers for antithrombin therapy has been of great interest since the earliest SELEX studies. To date, a number of SELEX experiments have been performed to obtain thrombin-binding aptamers (TBAs) using natural or modified nucleic acid libraries (Table 2) [63–69]. 

A minimized 20 mer RNA aptamer reported by Kubik et al. binds to human thrombin with a *K*_*d*_ value of 9.3 ± 1.0 nM. White et al. reported a minimized 25 mer RNA aptamer with superior binding affinity (*K*_*d*_ = 0.54 ± 0.1 nM), which was recovered by a method named toggle SELEX. A modified RNA aptamer comprising all four 4′-thionucleotides (A, G, C, U) was obtained with a *K*_*d*_ value of 7.2 nM. Another type of modified TBA consists of natural 2′-deoxy purine nucleotides (A, G) and 2′-OMe pyrimidine nucleotides (C, U), having libraries constructed with Y639F mutant T7 RNA polymerase, exhibiting a binding affinity in the nanomolar range (*K*_*d*_ = 26 nM). Minimized 15 mer and 29 mer DNA aptamers were reported by Bock et al. and Tasset et al., respectively; their respective *K*_*d*_ values were 113 ± 20 nM and 0.5 nM. A modified DNA aptamer containing 5-(1-pentynyl)-2′-dU exhibited weaker binding affinity with a *K*_*d*_ value of 400 nM. However, the experimental data indicated that these chemical modifications did not result in superior binding affinity.

Two electropositive domains (i.e., exosite-1 and exosite-2) that are displayed on the surface of thrombin are known to be TBA-binding sites [71]. Binding to these domains, which are distal from the catalytic site, could affect enzyme activity. The 15 mer DNA aptamer formed a two-tiered G-quadruplex and recognized exosite-1 via van der Waals forces and hydrogen bonding [72]. Although it was considered that the 29 mer DNA aptamer forms a two-tiered G-quadruplex containing structure based on its primary sequence, its binding site was found to be exosite-2 [73].

Natural RNA aptamers form hairpin structures and also bind to exosite-2. The 4′-thio TBA and the pentynyl TBA were predicted to have hairpin structures. With regard to the 2′-OMe TBA, the formation of potential stacked G-quartets was suggested by its multiple contiguous guanines. The binding sites for these modified TBAs have not been thoroughly investigated. Inferences based on NMR and X-ray structural studies of natural RNA/DNA aptamer-thrombin complexes [74, 75] may lead to the determination of appropriate modifications that are effective for improving affinity. 

## 4. Recent Advances

There are many successful examples of postmodifications, although, in many cases, unsatisfactory outcomes have resulted from the direct screening of modified RNA/DNA libraries. This indicates that chemical modifications should improve the binding properties if the proper functionalities are chosen. Recently, Vaught et al. demonstrated that screening using a modified DNA library containing 5-tryptaminocarbonyl-dU (TrpdU) could provide modified DNA aptamers that were specific for so-called “difficult protein targets,” which were previously intractable with SELEX using a natural DNA library (Table 3) [70]. A modified DNA aptamer involving TrpdU was found to bind tightly to necrosis factor receptor superfamily member 9 (TNFRSF9), a difficult protein target, with a *K*_*d*_ value of approximately 5 nM, whereas no DNA aptamer was recovered that could bind to TNFRSF9 from a natural DNA library using the same selection protocol.

Subsequently, significantly larger and more systematically conceived screenings were performed for 13 difficult protein targets using three different modified DNA libraries that contained TrpdU, 5-isobutylaminocarbonyl-dU (IbdU), and 5-benzylaminocarbonyl-dU (BndU), as well as a natural DNA library [76]. All screenings for these 13 protein targets were successful when a library containing TrpdU was used, whereas five and seven screenings failed when libraries containing IbdU and BndU, respectively, were used. No enrichment was observed in any of the screenings for these targets using a natural DNA library. 

The apparent *K*_*d*_ values of the enriched TrpdU-containing libraries were between several ten's of pM and several nM, which meant that sufficient enrichments of active species were achieved; some isolated aptamers exhibited extremely high-binding affinities at the level of several pM although the lowest *K*_*d*_ values differ with protein targets. These modified DNA aptamers were named Slow Off-rate Modified Aptamers (SOMAmers) because they were selected so as to have slow dissociation rates as a characteristic feature of their binding kinetics, which could improve binding specificity. SOMAmer technologies have enabled to expand over protein targets for which SELEX using natural RNA/DNA libraries did not yield high-affinity aptamers, which apparently illustrates the superiority of these chemical modifications.

During the preparation of modified DNA libraries for SOMAmer selection, *KOD XL* was used (essentially the same as *KOD Dash*). *KOD XL* comprises a mixture of approximately 2 : 98 of wild-type *KOD* DNA polymerase and *KOD(exo-)* DNA polymerase, which has no or less 3′,5′ exonuclease activity. We have focused on the high-catalytic activity and high fidelity of *KOD* DNA polymerase and its variants and first demonstrated its utility and application to PCR and SELEX involving modified nucleotides [25, 37, 56]. Recently, in our collaboration with Toyobo Co., Ltd., the catalytic properties of eight new *KOD* DNA polymerase variants in modified nucleotide polymerizations were assessed using base-/sugar-modified nucleoside triphosphates [77]. Among these *KOD* variants, one achieved efficient successive incorporation of bridged nucleotides with a 2′-ONHCH_2_-4′ linkage; this is much more bulky but exhibits far superior biostability than the prototype BNA/LNA. 

Thus, to expand the functional repertories of chemical modifications, both exploring favorable combinations of polymerases and substrate triphosphate analogs and genetically modifying polymerases have been performed. Developing highly efficient enzymatic polymerizations of artificially designed nucleic acid analogs with odd chemical structures, such as a bridged nucleic acid (BNA) [45], a glycol nucleic acid (GNA) [40], a peptide nucleic acid (PNA) [78], and a phosphorodiamidate morpholino oligomer (PMO) [79], would be expected to provide not only greatly enhanced affinities, specificities, and biostabilities but could also unexpected new functions for aptamers. Very recently, Pinherio et al. have developed evolved polymerases by a selection strategy named compartmentalized self-tagging (CST) [80], which is an improved methodology of compartmentalized self-replication (CSR) [81]. Using the evolved polymerases, six artificial biopolymers, 1,5-anhydrohexitol nucleic acid (HNA) [22], cyclohexenyl nucleic acids (CeNA) [36], arabinonucleic acid (ANA) [23], 2′-fluoroarabinonucleic acid (FANA) [24], *α*-L-threofuranosyl nucleic acid (TNA) [48], and BNA/LNA, were confirmed to be transcribed and reverse transcribed. Furthermore, HNA aptamers specific to HIV transactivating response (TAR) RNA and hen egg lysozyme (HEL) were successfully recovered by the traditional SELEX method using the acquired HNA polymerase although the binding affinities of the best aptamers were found to lie between the middle and high nanomolar range (*K*_*d*_ = 28 and 107 nM, resp.).

## 5. Conclusion

In over two decades of studies on nucleic acid aptamers, the effectiveness of employing chemically modified libraries for SELEX on binding properties was not evident until the development of SOMAmer, whereas that on biostability had been clearly evident in the early research era. Meanwhile, we could not conclusively prove that the chemical modifications may induce enhanced binding affinity, but we were successful in exemplifying functional expression, that is, high enantioselectivity, owing to the introduced foreign functionalities. Furthermore, first, we have focused on *KOD* DNA polymerase and its variants from various polymerases and demonstrated their feasibility for the direct screening of modified nucleic acid aptamers. Generally, the choice of polymerase has practical significance for our research purpose; not only catalytic efficiency but also the fidelity of polymerase greatly affects suitable enrichments during selection rounds because polymerase invariably causes misincorporation at a certain rate even with natural substrates. Despite the difficulties, it has been demonstrated that the successful engineering of polymerase can dramatically broaden the diversity of chemical structures for a selection library. 

One of the greatest concerns in the further development of this research is to gain systematic knowledge about the inter- and intramolecular interactions involving foreign functionalities for eventually deriving guidelines for the rational design of artificial specific binders. Nowadays, methodologies for the random screening of nucleic acid aptamers have become rapid and convenient, for example, capillary electrophoresis-SELEX [82] and microfluidic SELEX [83]; random screening can be implemented in systematic selections by using a varied modified library in order to address the challenging issue. 

## Figures and Tables

**Table 1:** Comparison of binding affinities in ATP-binding natural/modified nucleic acid aptamers.

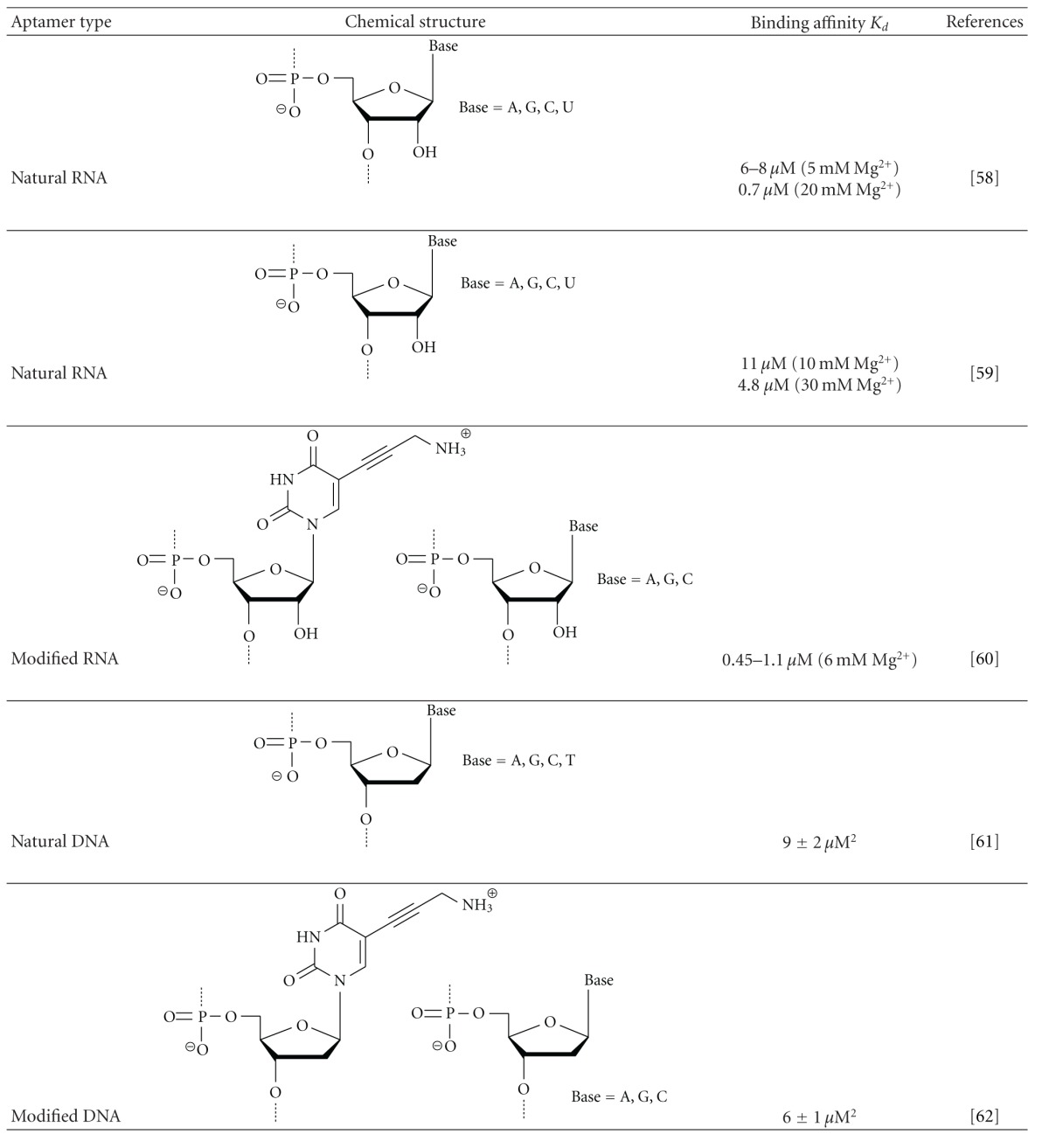

**Table 2:** Comparison of binding affinities in thrombin-binding natural/modified nucleic acid aptamers.

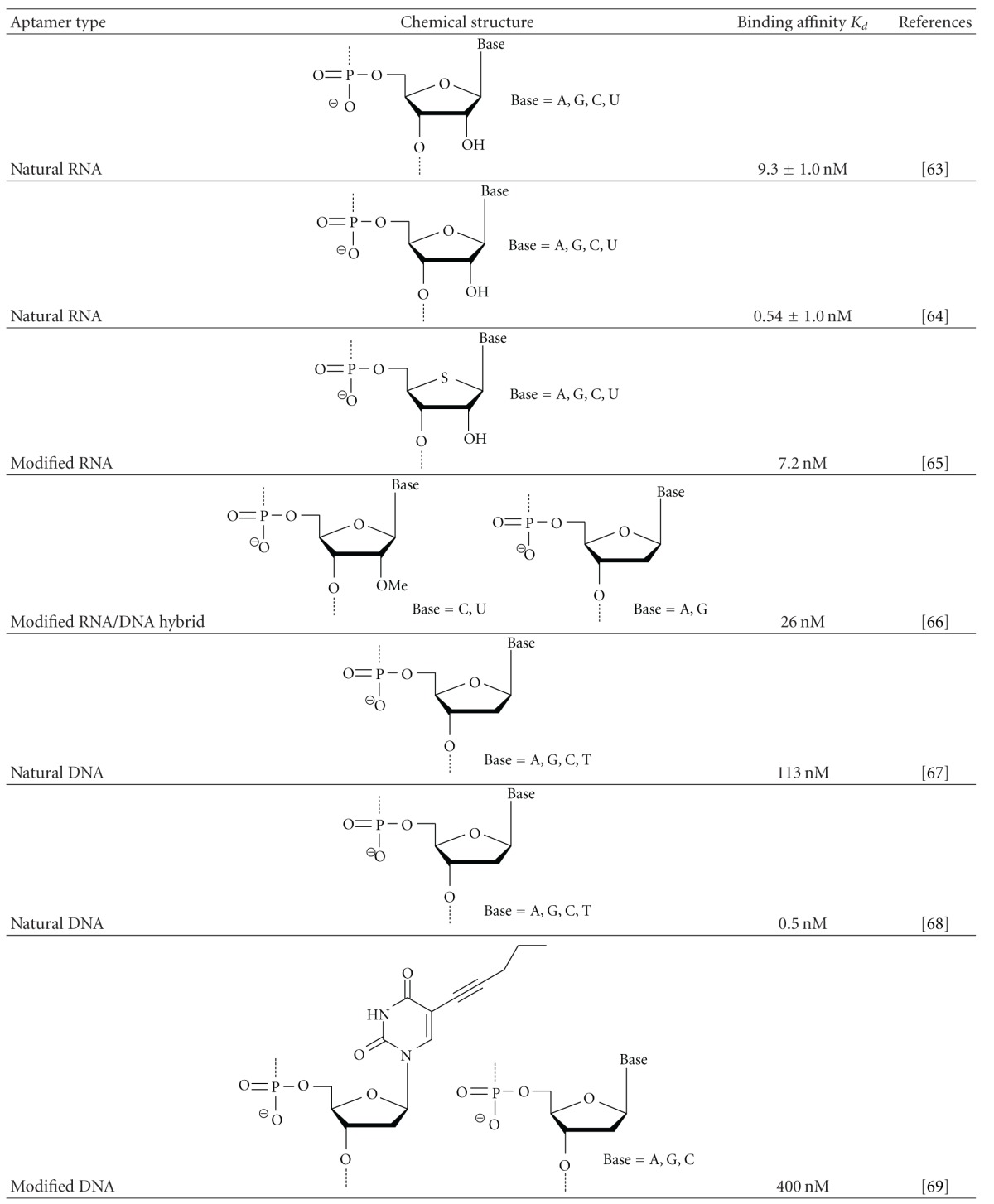

**Table 3:** Modified DNA aptamers containing C5-substituted thymidine having foreign functionalities, which were directly recovered from chemical libraries.